# Anxiolytic- and Antidepressant-Like Effects of Fish Oil-Enriched Diet in Brain-Derived Neurotrophic Factor Deficient Mice

**DOI:** 10.3389/fnins.2018.00974

**Published:** 2018-12-21

**Authors:** Juliane Zemdegs, Quentin Rainer, Cindy P. Grossmann, Delphine Rousseau-Ralliard, Alain Grynberg, Eliane Ribeiro, Bruno P. Guiard

**Affiliations:** ^1^Department of Physiology, Discipline of Nutrition Physiology, Universidade Federal de São Paulo, São Paulo, Brazil; ^2^Faculté de Pharmacie, Université Paris Sud, Université Paris-Saclay, Chatenay-Malabry, France; ^3^Centre de Recherches sur la Cognition Animale (CRCA), Centre de Biologie Intégrative (CBI), Centre National de la Recherche Scientifique, Université de Toulouse, Toulouse, France; ^4^INRA, Unité Mixte de Recherche BDR, ENVA, Université Paris Saclay, Jouy-en-Josas, France; ^5^INRA, Unité Mixte de Recherche 1154, Laboratoire Lipides Membranaires et Régulations Fonctionnelles du Coeur et des Vaisseaux, Jouy-en-Josas, France

**Keywords:** brain-derived neurotrophic factor (BDNF), neurobehavior, antidepressant, anxiolytic fish oil (n-3) fatty acids, serotonin

## Abstract

Despite significant advances in the understanding of the therapeutic activity of antidepressant drugs, treatment-resistant depression is a public health issue prompting research to identify new therapeutic strategies. Evidence strongly suggests that nutrition might exert a significant impact on the onset, the duration and the severity of major depression. Accordingly, preclinical and clinical investigations demonstrated the beneficial effects of omega-3 fatty acids in anxiety and mood disorders. Although the neurobiological substrates of its action remain poorly documented, basic research has shown that omega-3 increases brain-derived neurotrophic factor (BDNF) levels in brain regions associated with depression, as antidepressant drugs do. In contrast, low BDNF levels and hippocampal atrophy were observed in animal models of depression. In this context, the present study compared the effects of long-lasting fish oil-enriched diet, an important source of omega-3 fatty acids, between heterozygous BDNF^+/-^ mice and their wild-type littermates. Our results demonstrated lower activation of Erk in BDNF^+/-^ mice whereas this deficit was rescued by fish oil-enriched diet. In parallel, BDNF^+/-^ mice displayed elevated hippocampal extracellular 5-HT levels in relation with a local decreased serotonin transporter protein level. Fish oil-enriched diet restored normal serotonergic tone by increasing the protein levels of serotonin transporter. At the cellular level, fish oil-enriched diet increased the pool of immature neurons in the dentate gyrus of BDNF^+/-^ mice and the latter observations coincide with its ability to promote anxiolytic- and antidepressant-like response in these mutants. Collectively, our results demonstrate that the beneficial effects of long-term exposure to fish oil-enriched diet in behavioral paradigms known to recapitulate diverse abnormalities related to the depressive state specifically in mice with a partial loss of BDNF. These findings contrast with the mechanism of action of currently available antidepressant drugs for which the full manifestation of their therapeutic activity depends on the enhancement of serotoninergic and BDNF signaling. Further studies are warranted to determine whether fish oil supplementation could be used as an add-on strategy to conventional pharmacological interventions in treatment-resistant patients and relevant animal models.

## Introduction

Major depressive disorder (MDD) is an important public health concern worldwide. The lifetime prevalence of MDD is nowadays 15–20% of the population, and is expected to become the second most prevalent cause of illness-induced disability by 2020 (Lecrubier, 2001). These epidemiological data prompt research to identify the cellular and molecular mechanisms underpinning these mental disorders and to develop innovative treatments with better therapeutic effects than currently available medications. Indeed, despite their therapeutic activity, antidepressant drugs, including selective serotonin reuptake inhibitors (SSRIs), alleviate depression symptoms in only a limited percentage of patients, and remain insufficiently effective in treatment responders (Hamon and Blier, 2013).

Omega-3 polyunsaturated fatty acids (PUFAs) deficiency has been associated with several pathologies such as mood disorders, cardiovascular diseases, and stroke (Hibbeln et al., 2006). Mammals are unable to synthesize omega-3 and its supply depends on dietary intake. Fish oils represent the main source of omega-3 PUFAs [(i.e., eicosapentaenoic (EPA) and docosahexaenoic (DHA)] (Calder, 1998). Interestingly, it has been reported that depressed patients display low plasma and brain levels of omega-3 PUFAs (McNamara et al., 2007; Lin et al., 2010). Such deficits were also found in other populations with mental disorders: e.g., lower DHA and total omega-3 PUFAs in postpartum depression (De Vriese et al., 2003) and lower DHA in bipolar disorders (Chiu et al., 2003). Conversely, multiple sources of evidence suggested that consumption of omega-3 PUFAs produces antidepressant activity in patients with MDD (Peet et al., 1998; Marangell et al., 2003; Silvers et al., 2005; Freeman et al., 2006; Lin and Su, 2007; Owen et al., 2008; Su et al., 2008) or bipolar disorders (Montgomery and Richardson, 2008). A recent meta-analysis also revealed a beneficial overall effect of omega-3 PUFAs in patients under antidepressant drugs treatment (Mocking et al., 2016), suggesting that supplementation with these fatty acids could be used as an “add-on” strategy to reduce treatment resistance, and potentiate treatment response (Peet and Horrobin, 2002; Jazayeri et al., 2008; Gertsik et al., 2012). Consistent with these clinical studies, research in rodents showed that omega-3 PUFAs elicits a robust anxiolytic-like activity in the elevated plus maze (EPM) (Pérez et al., 2013) and an antidepressant-like activity in the forced swim and tail suspension tests (Blondeau et al., 2009; Venna et al., 2009; Moranis et al., 2012; Park et al., 2012; Vines et al., 2012). Moreover, omega-3 PUFAs were shown to improve anxiety-like and depressive-like phenotypes in various animal models of depression (Pérez et al., 2013; Pudell et al., 2014; Tang et al., 2015; Wu et al., 2016) and their combination with SSRIs appeared to be more effective than antidepressant drugs alone for reducing depression-like behaviors (Lakhwani et al., 2007; Laino et al., 2010; Able et al., 2014).

Antidepressant drugs activity is associated with the stimulation of brain serotonergic neurotransmission (Gardier et al., 1996) accompanied with an enhancement of adult hippocampal neurogenesis. On the contrary, disruption of hippocampal neurogenecis prevents the behavioral effects of various classes of antidepressant in mice (Schmidt and Duman, 2007). A number of factors have been proposed to participate in adult hippocampal neurogenesis and SSRI response including Brain-Derived Neurotrophic Factor (BDNF) (Nibuya et al., 1995). A single bilateral infusion of BDNF into the dentate gyrus of hippocampus produced antidepressant-like effects in naive mice (Deltheil et al., 2009) or in animal models of depression such as the learned helplessness (Shirayama et al., 2002). Interestingly, in heterozygous BDNF^+/-^ mice or in inducible BDNF KO lines of mice, deletion of BDNF in adults does not impact on depression-like behavior evaluated in the forced swim test (FST) (MacQueen et al., 2001; Saarelainen et al., 2003; Monteggia et al., 2007). However, these mutants display signs of antidepressant drugs resistance, notably at the behavioral and neurochemical levels (Saarelainen et al., 2003; Monteggia et al., 2004; Daws et al., 2007; Monteggia et al., 2007; Guiard et al., 2008; Ibarguen-Vargas et al., 2009). In an attempt to clarify the relationship between BDNF and the serotonergic system, alterations in behaviors regulated by serotonin such as hyperphagia and weight gain were demonstrated in BDNF^+/-^ mice (Lyons et al., 1999). BDNF^+/-^ mice also exhibit accelerated age-related loss of serotonergic innervation to the hippocampus (Lyons et al., 1999; Luellen et al., 2007) and increased expression of 5-HT transporter (Guiard et al., 2008). The latter effects likely contribute to dampen serotonergic neurotransmission (Siuciak et al., 1996; Mamounas et al., 2000) and strongly suggest that normal BDNF signaling is essential for antidepressant efficacy in mice.

Interestingly, the time course of omega-3 PUFAs-induced antidepressant-like effects in rodents is compatible with molecular and morphological changes taking place in the hippocampus. In particular, it has been reported that prolonged omega-3 PUFAs exposure stimulated BDNF expression and adult hippocampal neurogenesis in mice (Wu et al., 2004; Rao et al., 2007; Blondeau et al., 2009; Venna et al., 2009). In this context, the present study was designed to determine to what extent fish oil-enriched diet containing omega-3 PUFAs influence serotonergic tone and markers of hippocampal plasticity in BDNF^+/-^ mice and their wild-type littermates. Using behavioral paradigms assessing anxiolytic/antidepressant-like activities, we also examined whether fish oil-enriched diet represents an alternative therapeutic strategy to currently available antidepressant drugs in BDNF^+/-^ mice.

## Materials and Methods

### Animals and Dietary Treatment

**FIGURE 1:**
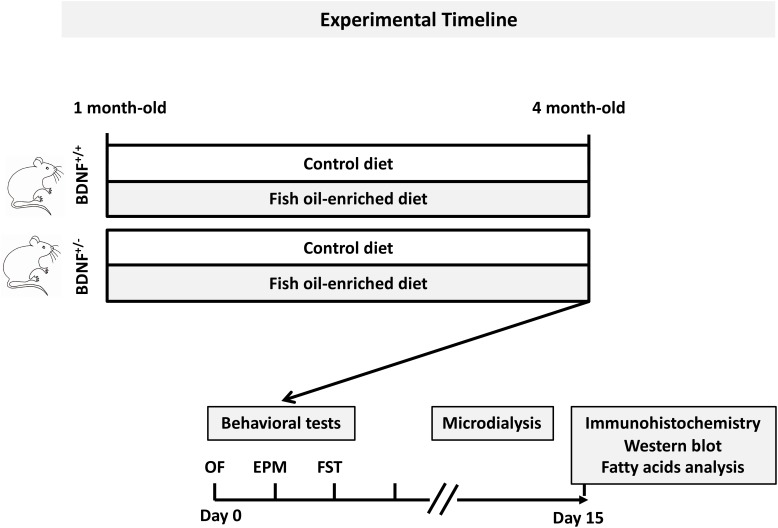
General procedure and experimental groups. Mice from each genotype were fed a control or fish oil-enriched diet for 12 weeks. *In vivo* procedures (i.e., behavioral and microdialysis experiments) were conducted while the animals were maintained under their respective diets. Given that it has been demonstrated that the interval between behavioral tests could be as little as 1 day, with a weak effect on overall performance (Paylor et al., 2006), in the present study, a 2-day recovery period between each test was provided. Moreover, to avoid the interference of behavioral testing on microdialysis experiments and notably the fact that the FST represents a strong stressor for the animals, the collection of dialysate samples was conducted 10 days after the behavioral assessment. The next day, animals were euthanized for immunohistochemistry, Western-blot and biochemical analyses.

Experiments were performed in accordance with the European Union (86/609/EEC) and the French National Committee of Ethics (87/848) policies regarding the care and use of laboratory animals. BDNF^+/-^ mice and their wild-type littermates initially bred on a mixed S129/Sv x C57BL/6 genetic background (Korte et al., 1995) were backcrossed to 129Sv strain, mated and raised at the animal facility of the *Université Paris-Sud* (Châtenay-Malabry, France) or at the *Universidade Federal de São Paulo* (Sao Paulo, Brazil). One-month-old male mice were genotyped by polymerase chain reaction and were randomly assigned to receive either a control diet or a fish oil-enriched diet for 12 weeks. Diets were prepared according to the recommendations of the American Institute of Nutrition (AIN-93) for rodents (Reeves, 1997) and were isocaloric and normolipidic, i.e., diets had identical energy and lipid content. The source of fat was soybean oil in the control diet and fish oil (Sigma-Aldrich, St. Louis, MO, United States). Both diets met the minimum suggested requirement for rodents of 2 g/kg diet of alpha-linolenic acid (ALA). The fatty acids composition of diets is depicted in Supplementary Table S1. Animals were housed in groups of five mice per cage under standard conditions (12:12 h light-dark cycle, 22 ± 1°C ambient temperature, 60% relative humidity), with *ad libitum* access to food and water. Experimental timeline is depicted in Figure 1. Procedures were conducted in conformity with the institutional guidelines in compliance with national and policy (Council directive #87–848, October 19, 1987, Ministère de l’Agriculture et de la Forêt, Service Vétérinaire de la Santé et de la Protection Animale, permission #92.196).

### Fatty Acids Analysis in the Hippocampus

Mice were killed by cervical dislocation. The brains were withdrawn and rinsed in saline (NaCl 0.9%). The hippocampi were finely dissected, weighed and stored at -80°C in CHCl3-MeOH (v/v, 2/1) to further determine its fatty acids profile. The lipids were extracted with chloroform/methanol (2/1), according to an adaptation of the method previously described (Rousseau et al., 2003). The phospholipids (PL) were separated from non-phosphorous lipids on silica acid cartridges. After the separation, the phospholipid fractions, mostly representative of the membranes, were transmethylated with boron trifluoride methanol 7% (Sigma-Aldrich, Saint Quentin Fallavier, France). The methyl esters of phospholipid fatty acids were analyzed by gas chromatography coupled to FID (Auto Sampling 8410 Gas Chromatograph 3900; Varian, Les Ulis, France) on an Econo-Cap EC-WAX capillary column (30-m, 0.32-mm internal diameter, 0.25-μm Film, ref 19654, ALLTECH Associates Inc., Templemars, France), using heptadecanoic acid (margaric acid, C17:0) as internal standard. Fatty acid composition was expressed as the percentage of total fatty acid weight. Desaturase activity was estimated according to Warensjo et al. (2009).

### Intracerebral Microdialysis for the Determination of Hippocampal Extracellular Serotonin (5-HT) Levels

Under anesthesia (chloral hydrate, 400 mg/kg, i.p.), mice were stereotaxically implanted with concentric microdialysis probes (active membrane length: 2.0 mm, molecular weight cut-off: 4.5 kD) in the ventral hippocampus (coordinates in mm from bregma: AP: -3.4, L: ± 3.4, V: 4.0). The next day, mice were connected to a swivel system and the probes were connected to a microinjection pump, allowing a continuous perfusion of artificial cerebrospinal fluid (composition: NaCl 147 mM, KCl 3.5 mM, CaCl2 1.26 mM, MgCl2 1.2 mM, NaH2PO4 1.0 mM, NaHCO3 25.0 mM; pH 7.4 ± 0.2) at a flow rate of 1.5 μl/ min. A 2 h-perfusion was performed to allow stabilization of 5-HT concentrations and microdialysis samples were then collected every 15 min. Microdialysates were kept at -80°C until analysis of 5-HT content by high performance liquid chromatography (HPLC) coupled to an amperometric detector (VT03; Antec Leyden, Netherlands). The amounts of 5-HT in microdialysates (19 μl) were calculated by measurement of peak heights relative to external standards. The limit of sensitivity for 5-HT was ∼ 0.5 fmol/sample (signal-to-noise ratio = 2).

### Western-Blot Analyses of Serotonin Transporter (SERT) and TrkB-BDNF Signaling Pathway

The hippocampi were homogenized in lysis buffer (1% Triton X-100, 100 mM Tris-HCl pH 7.4, 100 mM sodium pyrophosphate, 100 mM sodium fluoride, 10 mM EDTA, 10 mM sodium orthovanadate, 2.0 mM phenylmethylsulfonyl fluoride and 0.1 mg aprotinin/ml). Protein concentrations were determined using a commercial kit (BioAgency, Brazil). Equal amounts of proteins (50 μg) were loaded and separated on 10% SDS polyacrylamide gels and transferred onto nitrocellulose membranes (Amersham Biosciences, GE Healthcare, United States). Membranes were saturated with a blocking solution containing 1% BSA in TPBS (10 mM Tris, 150 mM NaCl and 0.02% Tween 20). Protein blots were incubated in 1% BSA in TPBS, overnight with the following primary antibodies: anti-phospho-Akt, anti-phospho-p44/p42 Erk, anti-SERT, and anti-alpha-tubulin. Primary antibodies were purchased from Cell Signaling (anti-phospho-Akt, anti phospho-p44/p42 Erk) or Santa Cruz Biotechnology (St. Louis, MO, United States) (serotoninergic and alpha-tubulin). After washing, membranes were incubated with the appropriate HRP-conjugated secondary antibodies (Sigma-Aldrich, St. Louis, MO, United States). Staining was revealed using the ECL-Plus Western blotting detection system (Thermo Scientific, Rockford IL, United States). Chemiluminescence was quantified by Scion Image software. After each revelation, membranes were incubated in stripping solution (62.6 mM Tris-HCl, 2% SDS, 100 mM *b*-mercaptoethanol, pH 6.8) for 30 min at 45°C and reblotted. Results are presented as the ratio of the protein, or phosphoprotein levels, to alpha-tubulin and are expressed as a percentage of the controls (wild-type under the control diet).

### Immunohistochemistry to Assess Adult Neurogenesis

#### 5-Bromo-2-Deoxyuridine (BrdU) Injection and Brain Preparation

A new cohort of mice was used for adult neurogenesis experiments. Just before the beginning of the dietary treatment, mice received i.p. injections of 5-bromo-2-deoxyuridine BrdU (150 mg/kg; 2 times/day) dissolved in saline (0.9% NaCl) for 4 days. After 12 weeks of dietary treatment, mice were deeply anesthetized with ketamine and transcardially perfused with 0.9% sodium chloride followed by 4% paraformaldehyde (PFA) in 0.1 M phosphate saline buffer (PBS). Brains were removed and postfixed overnight in 4% PFA at 4°C. Brains equilibrated in 30% sucrose 0.1 M phosphate buffer were embedded in Tissue-Tek OCT (Sakura, United States) and frozen. Coronal 40 μm-thick sections were obtained with a cryostat (Leica, Bensheim, Germany) and stored in cryoprotectant at -20°C until use.

#### BrdU Immunohistochemistry

One in six series of coronal sections (spaced 240 μm) throughout the rostrocaudal extent of the hippocampus was used for BrdU staining to evaluate new cell survival. Free-floating brain sections were rinsed in PB containing 0.9% NaCl and 0.25% Triton X-100 (PBST) before inactivation of endogenous peroxidases with 3% H_2_O_2_ in 10% methanol in PBS. Sections were incubated in 2N HCl in PBST for 50 min to denature DNA and then neutralized in 0.1M borate buffer (pH 8.5). Sections were then blocked in PBST containing 5% normal goat serum for 60 min, followed by overnight incubation in primary antibody monoclonal rat anti-BrdU (1:400; OBT-0030, Harlan Seralab, Loughborough, United Kingdom) in PBST with 0.1% sodium azide containing 5% normal goat serum. After incubation in goat anti-rat-biotinylated antibody (1:100, BA9400 Vector) for 1 h at room temperature, sections were incubated in the avidin-biotin complex (1:400 in PBS-T; Vector Laboratories ABC Elite Kit) and staining was visualized with DAB-Ni.

#### Doublecortin Immunohistochemistry

One in twelve series of coronal sections (spaced 480 μm) of the rostrocaudal extent of the hippocampus was used for doublecortin (DCX) staining to evaluate maturation of newborn neurons. Sections were incubated in 0.1M phosphate buffered saline with 0.5% Triton X-100 and 10% normal donkey serum (NDS), followed by goat anti-doublecortin primary antibody (1:500; Santa Cruz Biotechnology, SC8066, Santa Cruz, CA, United States) in TBS/Tx/NDS for 24 h at 4°C. Sections were then incubated in biotinylated donkey anti-goat secondary antibody (1:500; Jackson ImmunoResearch, West Grove, PA, United States) in TBS/NDS for 1 h at room temperature, followed by a 1 h amplification step using an avidin-biotin complex (Vector Laboratories ABC Elite Kit) and diaminobenzidine (DAB; Vectastain DAB Kit) as previously described (Quesseveur et al., 2013).

#### Quantification of Immunoreactive Cells

Slides were coded before analysis; the experimenter was blind to genotype and diet until all samples were counted. Quantification of BrdU-immunoreactive (BrdU+) and DCX-immunoreactive (DCX+) cells was conducted using Olympus BX51 microscope (Olympus Deutschland GmbH, Hamburg, Germany). The corresponding surface area of the granule cell layer (GCL) sampled for counting was measured using the Mercator stereology system (Explora Nova, La Rochelle, France). Density of positive cells was then calculated by dividing the number of positive cells by the GCL area sampled. Results were expressed as the number of positive cells/mm^2^.

### Behavioral Tests

Behavioral tests were performed between 9:00 and 11:00 am in a low light condition. Studies in animals are reported in accordance with the ARRIVE guidelines (McGrath et al., 2010). Thus, each mouse was subjected the open-field (OF), the EPM and the FST. This sequence was applied to minimize the impact of stress across tests and a 2-day recovery period between each test was provided (Figure 1). It is noteworthy that reducing the inter-test interval reduces the possible effect of dietary administration on tests.

*Open Field* was performed in Plexiglas setups (MED Associates, France) during a 30-min session. Entries count and total time in the center were measured by an automated system (MED Associated, France). Total ambulatory distance was also measured to ensure the absence of any locomotor effect of genotypes and/or diets.

*Elevated Plus Maze* was performed in a Plexiglas apparatus (MED Associates, France) during a 5-min session. Mice were placed in the center of the EPM facing an open arm and entries as well as time spent in the open and closed arms were measured by an automated system (ANY-maze, Stoelting Co., Wood Dale, IL, United States).

*Forced Swim Test* was performed in plastic buckets (20 cm diameter, 23 cm deep) filled up to two thirds with water at 23–25°C. FST was videotaped for a 6-min session period and the last 4 min were scored for active (climbing and swimming) and passive (immobility) behaviors by an experimenter blind to both genotypes and diets.

### Statistical Analysis

Statistical analysis was performed using GraphPad Prism software (Version 5, San Diego, CA, United States). Comparisons between groups were made using an analysis of variance (ANOVA), followed by Tukey’s *post hoc* analysis when warranted. Significance was set at *p* < 0.05.

## Results

### BDNF^+/-^ Mice and Their Wild-Type Littermates Fed a Fish Oil-Enriched Diet Similarly Incorporate Omega-3 PUFAs in Their Hippocampus

In the present study, two diets equivalent in total fat, protein, carbohydrate and caloric content were formulated. The control diet contained the fatty acid ALA, the precursor of omega-3 PUFAs, and the fish oil-enriched diet presented higher levels of EPA and DHA, two omega-3 PUFAs components (Supplementary Table S1). The fish oil-enriched diet increased levels of omega-3 PUFAs [*F*_(3,10)_ = 83.1; *p* < 0.001] and decreased the ratio omega-6 to omega-3 [*F*_(3,10)_ = 568.2; *p* < 0.001] in the hippocampus of both wild-type and BDNF^+/-^ mice. Hence, the partial genetic inactivation of BDNF did not prevent the incorporation of omega-3 fatty acids into hippocampal phospholipid membranes (Table 1).

**Table 1:** Fatty acids profile (% of total fatty acids) of hippocamrpus membrane phospholipids of wild-type and BDNF^+/-^ mice treated with control or fish oil-enriched diet.

	Wild-type mice		BDNF^+^/^-^mice	
	Control diet	Fish oil-enriched diet	Control diet	Fish oil-enriched d
**Fatty acids**				
C14:0	0.83 ± 0.02	0.95 ± 0.02	0.98 ± 0.21	0.79 ± 0.03
C16:0	23.17 ± 0.05	23.06 ± 0.12	15.34 ± 5.27	22.79 ± 0.17
C18:0	21.27 ± 0.05	20.78 ± 0.15	24.38 ± 1.87	20.60 ± 0.21
**Σ SFA**	**46.03 ± 0.01**	**45.58 ± 0.23**	**41.45 ± 3.27**	**45.02 ± 0.41**
C16:1n-7	0.57 ± 0.00	0.76 ± 0.034	0.933 ± 0.354	0.756 ± 0.046
C16:1cis-9	0.57 ± 0.02	0.64 ± 0.02	0.65 ± 0.12	0.62 ± 0.01
C18:1cis-9	14.41 ± 0.12	16.27 ± 0.16	15.87 ± 1.22	16.64 ± 0.13
**Σ MUFA**	**20.90 ± 0.19**	**22.93 ± 0.26**	**23.43 ± 2.08**	**23.34 ± 0.19**
C18:2 n-6 (LA)	1.44 ± 0.11	0.88 ± 0.03***	1.65 ± 0.1	0.68 ± 0.02***
C20:4 n-6 (AA)	12.20 ± 0.00	8.62 ± 0.10***	12.91 ± 0.57	8.45 ± 0.07***
C18:3 n-3 (ALA)	0.04 ± 0.00	0.17 ± 0.04	0.04 ± 0.01	0.04 ± 0.00
	0.03 ± 0.00	0.57 ± 0.05***	0.03 ± 0.01	0.63 ± 0.09***
C20:5 n-3 (DPA)				
	0.24 ± 0.02	0.67 ± 0.02***	0.23 ± 0.02	0.74 ± 0.02***
C22:5 n-3 (EPA)				
	14.49 ± 0.10	17.33 ± 0.24***	15.33 ± 0.30	17.62 ± 0.20***
C22:6 n-3 (DHA)				
***Σ* PUFAs**	**32.91 ± 0.04**	**30.95 ± 0.35^∗∗∗^**	**34.36 ± 1.78**	**30.96 ± 0.29^∗∗∗^**
	17.42 ± 0.04	11.77 ± 0.13***	18.50 ± 0.87	11.58 ± 0.07***
*Σ* PUFAs n-6				
*Σ* PUFAs n-3	15.13 ± 0.15	19.18 ± 0.21***	16.10 ± 0.30	19.38 ± 0.28***
	1.15 ± 0.01	0.61 ± 0.0***	1.15 ± 0.03	0.60 ± 0.01***
n-6/n-3				
**Desaturase activity**				
Δ9 16:1/16:0	0.025 ± 0.00	0.033 ± 0.00	1.244 ± 1.219	0.033 ± 0.002
Δ9 18:1/18:0	0.673 ± 0.008	0.783 ± 0.012***	0.654 ± 0.02	0.808 ± 0.016**
Δ6 20:3/18:2	0.343 ± 0.01	0.433 ± 0.027***	0.299 ± 0.021	0.519 ± 0.019**
Δ5 20:4/20:3	25.55 ± 0.43	22.847 ± 0.48	26.13 ± 0.99	24.21 ± 0.81


*SFA, saturated fatty acids; MUFA, monounsaturated fatty acids; PUFAs, polyunsaturated fatty acids; LA, linoleic acid; AA, arachidonic acid; ALA, alpha-linolenic acid; EPA, eicosapentaenoic acid; DHA, docosahesaenoic acid; DPA, docosapentaenoic acid. Σ SFA: sum of C14:0 + C15:0 + C16:0 + C18:0 + C20:0 + C22:0 + C23:0 + C24:0. Σ MUFA: sum of C14:1 n-9 + C15:1 n-9 + C16:1 n-7 + C16:1 n-9 + C16:1 n-11 + C18:1 n-7 + C18:1n-9 + C20:1 n-7 + C20:1 n-9 + C20:1 n-11 + C22:1 n-9. Σ PUFAs: sum of C18:3 n-3 + C18:4 n-3 + C20:3 n-3 + C20:4 n-3 + C20:5 n-3 + C22:5 n-3 + C22:6 n-3 + C18:2 n-6 + C18:3 n-6 + C20:2 n-6 + C20:3 n-6 + C20:4 n-6 + C22:2 n-6 + C22:3 n-6 + C22:4 n-6 + C22:5 n-6. Σ n-3: sum of C18:3 n-3 + C18:4 n-3 + C20:3 n-3 + C20:4 n-3 + C20:5 n-3 + C22:5 n-3 + C22:6 n-3. Σ n-6: sum of C18:2 n-6 + C18:3 n-6 + C20:2 n-6 + C20:3 n6 + C22:4 n-6 + C22:2 n-6 + C22:3 n-6 + C22:4 n-6 + C22:5 n-6. ^∗∗^*p* < 0.01 and ^∗∗∗^*p* < 0.001: significantly different from the corresponding group of mice fed a control diet (*n* = 3/4 mice/group).*

### Erk Phosphorylation Is Reduced in the Hippocampus of BDNF^+/-^ Mice and Can Be Rescued by Fish Oil-Enriched Diet

**FIGURE 2:**
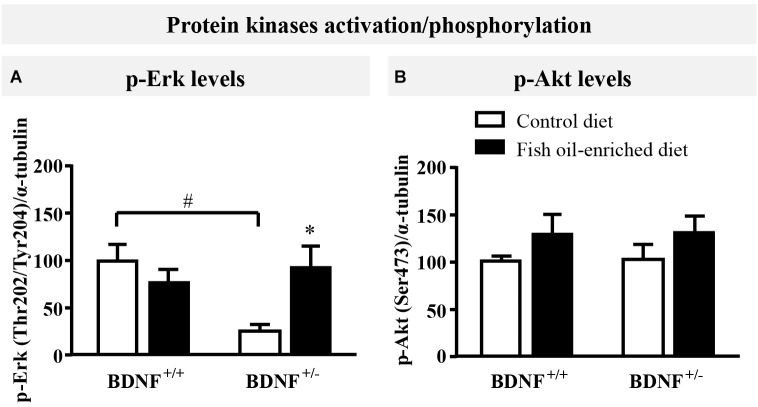
Fish oil-enriched diet increases p-Erk (Thr202/Tyr204) in the hippocampus of BDNF^+/-^ mice. **(A)** Densitometric quantification of immunoblot analysis from p-Erk (Thr202/Tyr204) (ANOVA: [*F*_(3,16)_ = 4.2; *p* = 0.02]) and **(B)** p-Akt (Ser473) (ANOVA: [*F*_(3,16)_ = 0.9; *p* = 0.4]) in the hippocampus of wild-type and BDNF^+/-^ mice fed a control (white bars) or fish oil-enriched diet (black bars) for 12 weeks. Data are expressed as means ± SEM of the ratio p-Erk/α-tubulin or p-Akt/α-tubulin (% of wild-type mice fed a control diet). ^∗^*p* < 0.05: diet effect, #*p* < 0.05: genotype effect (*n* = 5 mice/group).

Because initial studies demonstrated that forebrain BDNF mRNA and protein levels in BDNF^+/-^ mice were ≈50% of the wild-type (Ernfors et al., 1994; Korte et al., 1995; Kolbeck et al., 1999), the activation of ErK and Akt in the hippocampus was used in the present study as an indirect marker of changes in BDNF signaling (Schmidt and Duman, 2010; Quesseveur et al., 2013; Lepack et al., 2016). Under control diet, a significant reduction in p-Erk protein levels was unveiled in BDNF^+/-^ mice compared to wild-type littermates (*p* = 0.04, Figure 2A). Fish oil restored p-Erk protein to normal levels (*p* = 0.04) in BDNF^+/-^ mice, whereas it had no impact on p-Erk in wild-type animals (*p* = 0.7; Figure 2A). We also monitored the expression of p-Akt and found that partial BDNF depletion had no impact this parameter. Moreover, no significant effects were detected in either wild-type nor BDNF^+/-^ mice fed a fish oil-enriched diet [*F*_(3,16)_ = 0.9; *p* = 0.4; Figure 2B]. Altogether, these data support the fact that the partial BDNF depletion leads to impairment of Erk signaling pathway, a deficit which can be rescued by fish oil-enriched diet.

### Fish Oil-Enriched Diet Thwarts the Perturbation of Serotoninergic Neurotransmission Induced by BDNF Depletion in the Hippocampus

**FIGURE 3:**
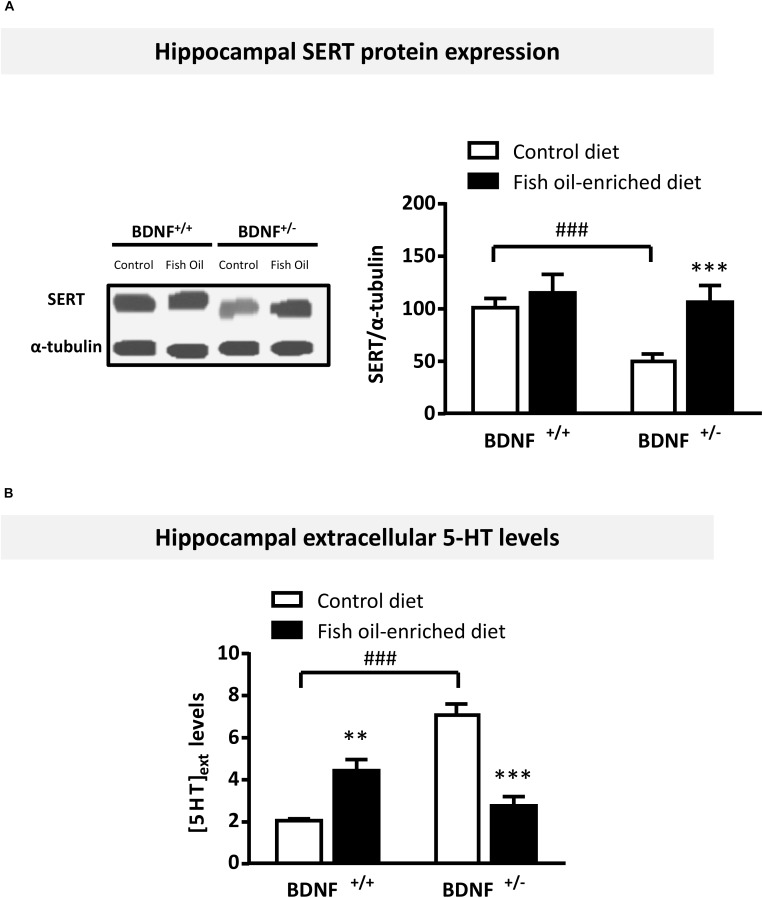
Fish oil-enriched diet decreases serotonergic tone in the hippocampus of BDNF^+/-^ mice. **(A)** Protein expression of the Serotonin Transporter (SERT). (Left panel) Representative blots from SERT. (Right panel) Densitometric quantification of immunoblot analysis from SERT (ANOVA: [*F*_(3,29)_ = 5.2; *p* = 0.002]) in the hippocampus of wild-type and BDNF^+/-^ mice fed a control (white bars) or fish oil-enriched diet (black bars) for 12 weeks. Data are means ± SEM of the ratio SERT/α-tubulin (% of wild-type mice fed a control diet). ^∗^*p* < 0.05: diet effect; #*p* < 0.05: genotype effect significantly different from wild-type mice fed a control diet (*n* = 8–9 mice/group). **(B)** Basal extracellular 5-HT levels ([5-HT]_ext_) in the hippocampus. Data are means ± SEM of basal [5-HT]_ext_ (fmol/19 μL) measured for a 60 min-period in the hippocampus of mice from both genotypes fed a control (white) or fish oil-enriched diet (black) for 12 weeks (ANOVA: [*F*_(3,25)_ = 67.23]; *p* < 0.001). ^∗∗^*p* < 0.01 and ^∗∗∗^*p* < 0.001: diet effect; ###*p* < 0.001: significantly different from wild-type mice fed a control diet (*n* = 6–9 mice/group).

We then sought to determine whether fish oil-enriched diet influenced hippocampal serotonergic tone by first assessing the expression of the 5-HT transporter SERT in the hippocampus of wild-type and BDNF^+/-^ mice. Under control diet, a significant decrease in SERT protein levels was observed in BDNF^+/-^ compared to wild-type mice (*p* = 0.015). Although fish oil had no effect on SERT protein levels in wild-type mice (*p* = 0.7), this diet rescued SERT protein to control levels in BDNF^+/-^ mice (*p* = 0.02; Figure 3A). In light of these findings, we tested the possibility that serotonergic tone might be differentially modified in wild-type and BDNF^+/-^ mice. Accordingly, under control diet a significant increase in extracellular 5-HT levels ([5-HT]_ext_) was detected in the ventral hippocampus of BDNF^+/-^ mice compared to their wild-type littermates (*p* < 0.001). Fish oil-enriched diet normalized this parameter in BDNF^+/-^ mice (*p* < 0.001; Figure 3B). These results indicate that the partial BDNF depletion is responsible for an enhancement of serotonergic tone in the hippocampus whereas fish oil-enriched diet restored normal [5-HT]_ext_ levels in BDNF^+/-^ mice by heightening SERT protein expression.

### Fish Oil-Enriched Diet Increases Densities of Immature Adult-Born Neurons in the Dentate Gyrus of BDNF^+/-^ Mice

**FIGURE 4:**
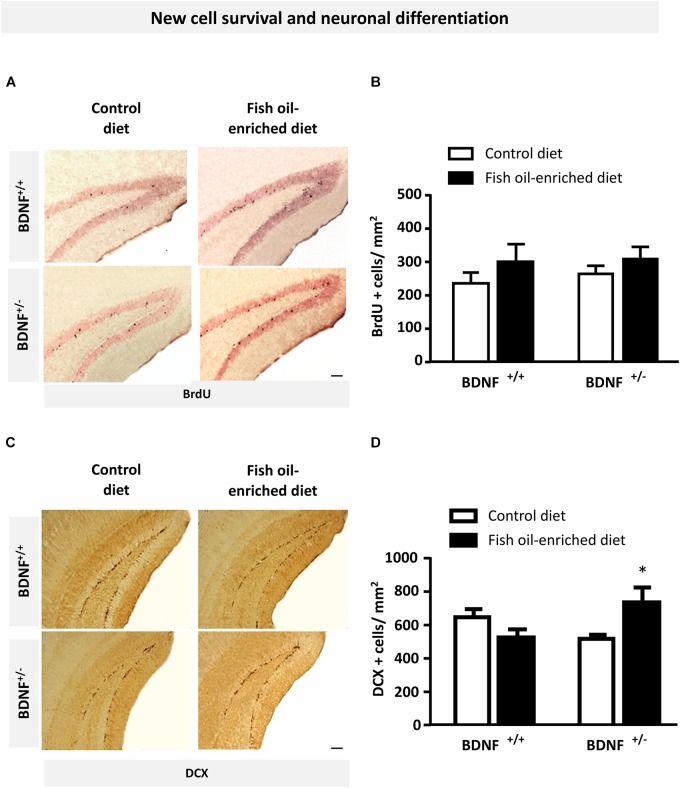
Fish oil-enriched diet does not affect cell survival but increases the density of immature neurons in the hippocampus of BDNF^+/-^ mice. **(A)** Representative images of the dentate gyrus after 5-bromo-2-deoxyuridine (BrdU) immunostaining in each experimental group. Scale bar: 50 μm. **(B)** Density of BrdU-labeled (BrdU+) cells 4 weeks after BrdU injection indicative of new cell survival. Data are means ± SEM of BrdU+ cell counts per mm^2^ (ANOVA: [*F*_(3,19)_ = 0.8; *p* = 0.4]; *n* = 5–6 mice/group) in the dentate gyrus of wild-type and BDNF^+/-^ mice fed a control (white) or fish oil-enriched diet (black) for 12 weeks. **(C)** Representative images of the dentate gyrus showing doublecortin-labeled (DCX+) cells in each experimental group. Scale bar: 50 μm. **(D)** Density of DCX+ cells in the dentate gyrus, indicative of the presence of immature neurons. Data are means ± SEM of DCX+ cell counts per mm^2^ (ANOVA: [*F*_(3,26)_ = 3.6; *p* = 0.025]) in the dentate gyrus of wild-type and BDNF^+/-^ mice fed a control (white) or fish oil-enriched diet (black) for 12 weeks. ^∗^*p* < 0.05: diet effect (*n* = 6–9 mice/group).

Because previous reports pointed out a role for 5-HT and BDNF signaling in the control of hippocampal plasticity, particularly regarding its ability to influence new cell survival and neuronal differentiation (Nibuya et al., 1995; Quesseveur et al., 2013), we examined the effects of fish oil-enriched diet on these parameters in wild-type and BDNF^+/-^ mice. To this end, we quantified BrdU- and DCX-labeled (BrdU+ and DCX+) cells in the dentate gyrus of mice from both genotypes. Densities of 12-week-old BrdU+ cells were not significantly different among experimental groups (Figures 4A,B), indicating that partial BDNF depletion has no long-term impact on hippocampal new cell survival. As regards the density of DCX+ cells, under control diet, neuronal differentiation was not different between wild-type and BDNF^+/-^ mice. Interestingly, although fish-oil diet failed to alter this parameter, a significant increase in the density of DCX+ cells was observed in BDNF^+/-^ mice (*p* = 0.05; Figures 4C,D). These results suggest that fish-oil enriched diet increases the pool of immature neurons in the dentate gyrus of BDNF^+/-^ mice.

### Fish Oil-Enriched Diet Exerts Antidepressant and Anxiolytic-Like Effects on BDNF^+/-^ Mice

Finally, we tested the effects of fish oil-enriched diet on mice from both genotypes submitted to behavioral paradigms designed to evaluate different symptoms of depressive state such as anxiety in the OF and EPM and despair in the FST.

**FIGURE 5:**
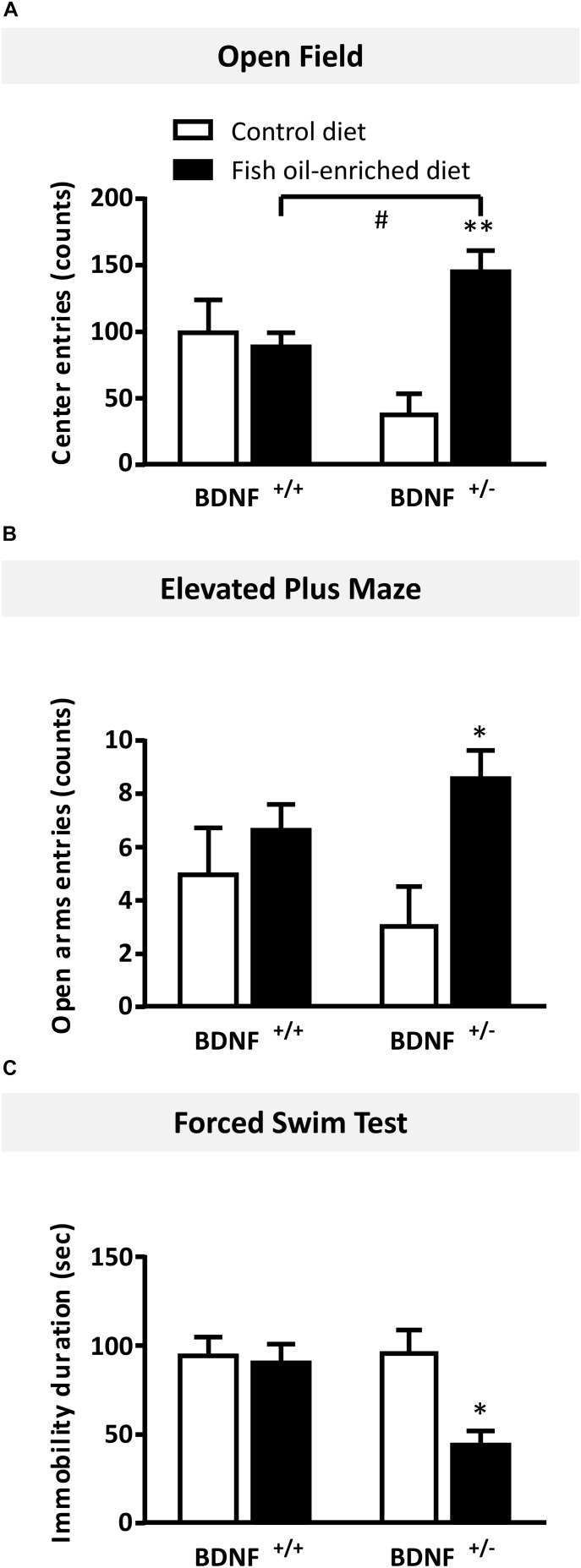
Fish oil-enriched diet induces anxiolytic- and antidepressant-like activities in BDNF^+/-^ mice. **(A)** Anxiety evaluated in the open field. Data are means ± SEM of the number of entries in the center of the arena (ANOVA: [*F*_(3,20)_ = 7.1; *p* = 0.002]). **(B)** Anxiety evaluated in the elevated plus maze.*(Data are means ± SEM of the number of entries in the open arms (ANOVA: [*F*_(3,20_ = 2.8; *p* = 0.11]). **(C)** Antidepressant-like activity evaluated in the forced swim test (FST). Data represent means ± SEM of the immobility time of wild-type and BDNF^+/-^ mice fed a control (white) or fish oil-enriched diet (black) for 12 weeks (ANOVA: [*F*_(3,20)_ = 7.1; *p* = 0.002]). ^∗^*p* < 0.05, ^∗∗^*p* < 0.01: diet effect; #*p* < 0.05: genotype effect (*n* = 6 mice/group).*

In the OF test, numbers of entries in the center were not statistically different between wild-type and BDNF^+/-^ mice fed a control diet (*p* = 0.06). In mice fed the fish oil-enriched diet, the number of entries (*p* < 0.01) was significantly increased in BDNF^+/-^ mice (*p* < 0.01), but not in wild-type littermates (*p* = 0.7; Figure 5A). Interestingly, similar results were obtained on the time spent in the center (Supplementary Figure S1A). To eliminate a putative bias, we verified that the locomotor activity was not different between groups (wild-type control diet: 3195 ± 461 cm during 30 min; BDNF^+/-^ control diet: 2694 ± 209 cm; wild-type fish oil-enriched diet: 2794 ± 305 cm and BDNF^+/-^ fish oil-enriched diet: 3201 ± 154 cm [F_(3,20)_ = 0.7; p = 0.5491]).

Likewise, in the elevated plus maze, under control diet, no differences were detected between BDNF^+/-^ and their wild-type littermates in the number of entries in the open arms (*p* = 0.4). However, in mice fed the fish oil-enriched diet, this parameter was significantly increased in BDNF^+/-^ mice (*p* < 0.01) but not in wild-type littermates (*p* = 0.4; Figure 5B). Again, these results are consistent with those obtained on the time spent in the open arms (Supplementary Figure S1B).

Finally, in the FST, under control diet, wild-type and BDNF^+/-^ mice displayed the same duration of immobility (*p* = 0.9). Fish oil significantly decreased the time of immobility in BDNF^+/-^ mice (*p* < 0.01), while it had no effect in wild-type mice fed a control diet (*p* = 0.8; Figure 5C).

Overall, these set of behavioral data indicate that fish oil-enriched diet elicited anxiolytic- and antidepressant-like activities specifically in BDNF^+/-^ mice.

## Discussion

The present study evaluated the effects of prolonged exposure to fish oil-enriched diet in wild-type and BDNF^+/-^ mice at the molecular, cellular and behavioral levels. BDNF^+/-^ mice offer a good model to study non-conventional therapeutic strategies for anxiety and depression since these mice are less prone to respond to currently available antidepressant drugs including SSRIs (Daws et al., 2007; Guiard et al., 2008; Ibarguen-Vargas et al., 2009). One of the most remarkable results obtained herein is that under control diet, the partial BDNF depletion produced significant changes in the hippocampus, including a decreased activation of the MAP kinase Erk along with an elevated serotonergic tone. However, these effects were not sufficient to impact cell survival and neuronal differentiation in the hippocampus and to impact anxio-depressive-like behaviors. In a marked contrast, long-term exposure to fish oil-enriched diet in BDNF^+/-^ but not in BDNF^+/+^ mice increased the activation of Erk and decreased serotonergic tone. These molecular changes were accompanied by an enhancement of neuronal differentiation along with reproducible anxiolytic responses and robust antidepressant-like effects. Together these results led us to envision that fish oil could exert its beneficial effects on mood specifically in patients displaying decreased BDNF signaling in the hippocampus.

The effects of fish oil-enriched diet, an omega-3 PUFAs source, on anxiety have been poorly documented. The limited data available in rodents show that supplementation of diet with omega-3 PUFAs favors anxiolytic-like activities (Pérez et al., 2013; Pudell et al., 2014), whereas their deprivation produces opposite effects (Lafourcade et al., 2011; Larrieu et al., 2012). The latter findings are not consistent with the present results since we failed to unveil beneficial effects of fish oil in wild-type BDNF^+/+^ mice submitted to the OF and EPM. One possible explanation for this relies on the fact that the control diet used in the present study was not deficient in omega-3. Indeed, the therapeutic benefits of fish oil supplementation have been found only in omega-3 deficient individuals, while those without baseline deficits were less prone to benefit from supplementation (Horrobin and Bennett, 1999). Alternatively, one would expect that fish oil-enriched diet specifically dampens anxiety under pathological conditions. In agreement with this hypothesis, evidence demonstrated that the anxiolytic-like effects of omega-3 PUFAs are detectable after acute or chronic stress (Harauma and Moriguchi, 2011; Mathieu et al., 2011; Ferreira et al., 2013). Of particularly interest in the context of the present study unveiling anxiolytic-like effects of fish oil in BDNF^+/-^ mice, it has been reported that such diet displays similar behavioral properties in bulbectomized rats, an animal model of depression also characterized by reduced hippocampal BDNF levels (Pudell et al., 2014). In keeping with the latter findings, omega-3 PUFAs supplementation was shown to improve social interaction in a strain of mice displaying a reduction of BDNF levels in various brain regions (Pietropaolo et al., 2014). Collectively, these results suggest that the down-regulation of BDNF may be a prerequisite for the manifestation of fish oil’s anxiolytic-like activity. These findings are important since previous studies reported that BDNF^+/-^ mice are not responsive to chronic imipramine treatment (Ibarguen-Vargas et al., 2009) or to acute paroxetine administration (Guiard et al., 2008). Hence, fish oil-enriched diet might be used either alone or as an add-on strategy to antidepressant drugs in treatment-resistant patients (Laino et al., 2010, 2014; Hou and Lai, 2016; Mayor, 2016).

To unravel the putative links between BDNF deficiency and behavioral effects of fish oil-enriched diet, we examined the functional activity of two proteins kinases in the hippocampus (i.e., Erk and Akt). Doing so, we observed that BDNF^+/-^ mice displayed a significant reduction in the level of Erk phosphorylation/activation whereas fish oil diet increased this deficit. Such reversal effect might have contributed, at least in part, to the anxiolytic properties of fish oil diet in these mutants. In support of this hypothesis, we recently demonstrated that an increase in hippocampal p-Erk correlates with a decrease in the latency to feed in the novelty suppressed feeding test (Quesseveur et al., 2015). Conversely, evidence showed that rats microinjected with a specific inhibitor of Erk in the hippocampus for seven consecutive days display anxiety-like behaviors in the open field and the elevated plus maze (Qi et al., 2009). Having shown that fish oil diet increases p-Erk and exert anxiolytic effects specifically in BDNF^+/-^ mice, we then explored to what extent the serotonergic system could be involved an additional and possible component in the behavioral characteristic of fish oil-enriched diet. Here, we report an increase in basal [5-HT]_ext_ in the ventral hippocampus of BDNF^+/-^ mice, which is normalized in response to fish oil-enriched diet. Interestingly, evidence indicated that an abnormally elevated 5-HT tone favors anxiety through the activation of specific post-synaptic 5-HT receptors including the 5HT_1A_, 5-HT_2A/C_ or 5-HT_3_ subtypes (Hamon, 1994). Although the impact of fish oil-enriched diet on hippocampal [5-HT]_ext_ levels remains elusive, a recent study pointed out that omega-3 PUFAs supplementation in diet increases tissue 5-HT contents in hippocampus and cortex associated to reduced 5-HIAA levels in 3 months-old rats (Vines et al., 2012). The latter findings are relevant in light of our microdialysis data since an accumulation of tissue (i.e., intracellular) 5-HT in fish oil fed animals could reflect lower [5-HT]_ext_, notably if the release process is dampened (Kodas et al., 2004). It is noteworthy that the putative inhibitory influence of fish oil-enriched diet on [5-HT]_ext_ levels in the hippocampus of BDNF^+/-^ mice may also result from mechanisms involving the tryptophan hydroxylase 2 (TpH2), the rate-limiting enzyme of 5-HT synthesis, and/or the monoamine oxydase (MAO), an enzyme important in the catabolism of 5-HT. However, the observations that omega-3 PUFAs enhance the expression of tryptophan hydroxylase-2 (TPH-2) (McNamara et al., 2009), while attenuating that of MAO-A/B in the brain (Delion et al., 1997; Chalon et al., 1998; Naveen et al., 2013) are not compatible with our neurochemical data. Because the serotonin transporter SERT is an alternative target through which 5-HT tone may be regulated, we studied this molecular element in the hippocampus. As previously described, we found that BDNF^+/-^ mice exhibited lower levels of SERT protein expression in the hippocampus (Guiard et al., 2008) thereby resulting in an elevated 5-HT tone. Fish oil-enriched diet restored normal hippocampal SERT expression, a process that contributed to normalize 5-HT tone in the hippocampus in BDNF^+/-^ mice and probably in other brain regions. Hence, we demonstrated that fish oil-enriched diet corrected SERT down-regulation directed at minimizing the basal hyperserotonergic phenotype reported in BDNF^+/-^ mice. It is noteworthy that increased anxiety-related behaviors were observed in adult SERT^-/-^ mice (Holmes et al., 2003; Kalueff et al., 2010; Sakakibara et al., 2014) which display spontaneous higher [5-HT]_ext_. A corollary of this observation is that a functional SERT is necessary to promote long-term fish oil-induced anxiolysis as reported herein. In humans, a short promoter variant in the SERT gene is linked to lower SERT expression, leading to a reduced 5-HT reuptake (Bengel et al., 1998; Murphy and Lesch, 2008). This short variant has also been associated with anxiety-related personality traits (Lesch et al., 1996; Hariri and Holmes, 2006; Canli and Lesch, 2007) and it would be relevant to determine whether long-term exposure to fish oil is effective in this specific population of patients. Again, fish oil-enriched diet had no effect on serotonergic activity in wild-type animals. This is likely due to the fact that these mice display normal levels of anxiety and 5-HT transmission at baseline.

As regards the antidepressant-like effects of fish oil in BDNF^+/-^ mice, it is difficult to envision that decreased immobility observed in the FST relates to the decreased serotonergic tone since an activation of 5-HT neurotransmission is required to hinder behavioral despair in this paradigm (Page et al., 1999). Alternative mechanisms are likely responsible for the antidepressant response induced by long-term exposure to fish oil-enriched diet. It is noteworthy that changes in hippocampal TrkB/BDNF transmission strongly influence MAP kinases signaling pathways, which in turn regulates depressive-related symptoms (Schmidt and Duman, 2010; Lepack et al., 2016). For example, interventions producing antidepressant-like effects such as electroconvulsive shocks or chronic administration of antidepressant drugs are generally associated with an up-regulation of BDNF and downstream signaling pathways in various brain regions including the hippocampus (Nibuya et al., 1995; Balu et al., 2008; Quesseveur et al., 2013). Based on this evidence, we can infer that the ability of fish oil to increase p-Erk levels in BDNF^+/-^ mice leading to a complete recovery of initial hippocampal levels played an important role in the induction of antidepressant-like effects. This is consistent with the observation that addition of DHA to rat primary culture of cortical astrocytes induced BDNF protein expression, an effect blocked by a MAPK inhibitor (Rao et al., 2007). Moreover, it is noteworthy that blunted Erk activation has been observed both in depressed patients and in relevant animal models of depression (Dwivedi et al., 2001; Feng et al., 2003; Gourley et al., 2008; Yuan et al., 2010) whereas inhibition of kinases such as MEK or Erk produced despair-like behaviors and prevented the antidepressant-like effects of SSRIs in rodents (Shirayama et al., 2002; Duman et al., 2007). The observation that fish oil-enriched diet had no effect on Erk activation in wild-type animals is consistent with the lack of effects of this diet on behavioral parameters. The putative enhancement of BDNF signaling, as suggested by Erk phosphorylation/activation in response to fish-oil enriched diet in BDNF^+/-^ mice, also draw our attention because MAP kinases play an important role in the regulation of adult hippocampal neurogenesis including the stimulation of proliferation/differentiation of neural progenitor cells (Lee et al., 2002; Sairanen et al., 2005; Li et al., 2008; Taliaz et al., 2010), the maturation of newborn neurons and their survival (Lee et al., 2002; Sairanen et al., 2005; Rossi et al., 2006; Wang et al., 2015). However, comparing densities of surviving BrdU+ cells, no differences were detected between wild-type and BDNF^+/-^ mice fed a control or a fish oil-enriched diet. However, from these results we cannot provide definitive conclusion on cell survival as long as the number of new generated cells is not assessed in all experimental groups. Given that some studies have described a neurogenic effect of omega-3 PUFAs through its ability to stimulate neuronal maturation (Grundy et al., 2014; McCall et al., 2015), we also quantified immature neurons, using DCX immunolabeling. Interestingly, we observed increased numbers of DCX+ cells in the dentate gyrus of BDNF^+/-^ mice fed a fish oil-enriched diet, suggesting that adult neurogenesis is impacted. Whether increased numbers of DCX+ cells reflect a preferential engagement of newborn cells toward a neuronal fate or a delay in terminal neuronal differentiation remains to be explored.

In an attempt to identify the putative beneficial effects of fish oil, we have to take into consideration the possibility that it might act by modulating inflammatory processes. Indeed, systemic administration of (LPS), widely used to create neuroinflammation, is known to precipitate depression-related behaviors in rodents (Dantzer et al., 2008) whereas evidence indicates that the antidepressant-like effects of fish-oil result, at least in part, from its ability to attenuate this state (Moranis et al., 2012; Delpech et al., 2015; Fourrier et al., 2017). A recent study reported that the TrkB agonist 7,8-dihydroxyflavone (7,8-DHF) reversed LPS-induced depression-like phenotype and morphological changes (i.e., spine density) in the mouse hippocampus (Zhang et al., 2014). These findings suggest that the enhancement of BDNF signaling could be a prerequisite to decrease neuroinflammation (Xu et al., 2017) and to promote the beneficial effects of fish oil in BDNF^+/-^ mice. Nevertheless, different reports indicated that BDNF^+/-^ mice are protected from inflammation not only in the whole brain (Javeri et al., 2010) but also in peripheral tissues such as the heart and the gut (Yang et al., 2010; Halade et al., 2013). Although these findings argue against the fact that BDNF^+/-^ mice could display hallmarks of neuroinflammation, further investigations are warranted to determine to what extent inflammatory processes such as increases in the expression of pro-inflammatory cytokines, activation of ubiquitous indoleamine 2,3-dioxygenase (IDO) or recruitment of microglial cells are altered in these mutants and whether fish-oil enriched diet positively reverberates on these specific markers in the hippocampus.

## Conclusion

**FIGURE 6:**
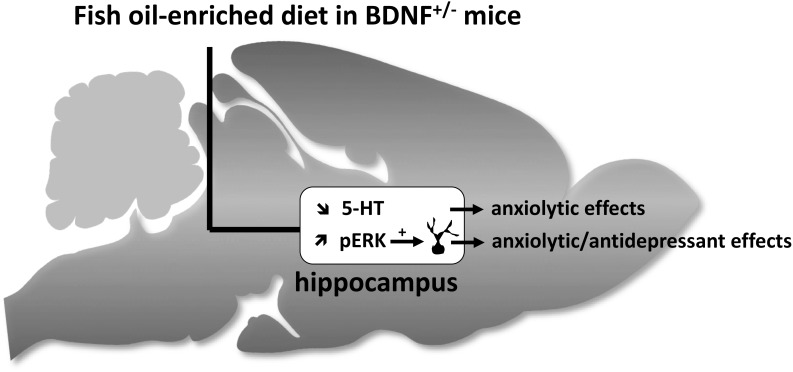
Schematic representation of hippocampal neurochemical, molecular and cellular targets of fish oil-enriched diet and its behavioral effects in BDNF^+/-^ mice. In BDNF^+/-^ mice, a decrease in the extracellular 5-HT concentrations and an increase in the level of Erk phosphorylation are observed in response to long-term exposure to a fish oil-enriched diet. These effects might have contributed to positively reverberate on anxiety and despair. However, because the enhancement of BDNF synthesis/release and related-signaling (Erk activation) in the hippocampus relies on the activation of local 5-HT tone, the results presented herein cannot draw a cause and effect relationship between BDNF signaling and 5-HT neurotransmission. We propose that the beneficial behavioral effects of fish-oil in BDNF^+/-^ mice involved two distinct mechanisms leading on one hand, to decrease extracellular 5-HT concentrations (favorable for anxiolysis) and on the other hand, to stimulate BDNF signaling and neuronal maturation (favorable for anxiolysis and antidepressant response).

Our data demonstrate that BDNF^+/-^ mice were more sensitive to the effects of fish oil-enriched diet than wild-type mice. As depicted in Figure 6, the present study strongly suggests that fish oil positively reverberates on emotionality through its ability to decrease hippocampal extracellular 5-HT levels and to increase the activation of Erk that might contribute by itself to stimulate neuronal plasticity. It should be borne in mind that fish-oil is a mix of omega-3 fatty acids including, for example, eicosapentaenoic acid (EPA), docosahexaenoic acid (DHA) or α-linolenic acid (ALA). Given that these components display distinct effects on behavioral paradigms assessing antidepressant-like activities (Jin and Park, 2015; Choi and Park, 2017), it would be interesting to precise which of them interfere specifically on neurobehavior. This will help optimize an “add-on” strategy based on the combination of fish oil and SSRI in animal models resistant to conventional monoaminergic antidepressant drugs. In particular, it will be interesting to determine the effects of fish oil-enriched diet in mice exposed to chronic stress (e.g., restraint stress, unpredictable chronic mild stress or even social defeat).

## Ethics Statement

The *in vivo* part of this study was conducted in 2013 and the procedures were conducted in conformity with the institutional guidelines in compliance with national and policy (Council directive #87–848, October 19, 1987, Ministère de l’Agriculture et de la Forêt, Service Vétérinaire de la Santé et de la Protection Animale, permission #92.196). The *in vitro* part of this study, not subjected to an ethical committee, was conducted between 2015 and 2017.

## Author Contributions

JZ conducted all the experiments in BDNF wild-type and mutant mice fed a fish oil diet and wrote the manuscript. QR and CG provided their assistance for the behavioral and Western-blot analyses. DR-R and AG prepared the fish oil diet and analyses. ER and BG were the principal investigators of this study and contributed to the analyses of the results.

## Conflict of Interest Statement

The authors declare that the research was conducted in the absence of any commercial or financial relationships that could be construed as a potential conflict of interest.

## Supplementary Material

Figure S1 Fish oil-enriched diet induces anxiolytic-like activities in BDNF^+/-^ mice. **(A)** Anxiety evaluated in the open field. Data are means ± SEM of the time spent in the center of the arena (ANOVA: [*F*_(3,20)_ = 7.1; *p* = 0.002]). **(B)** Anxiety evaluated in the elevated plus maze. Data are means ± SEM of the time spent in the open arms (ANOVA: [*F*_(3,20)_ = 2.8; *p* = 0.11]). ^∗^*p* < 0.05: significantly different from the corresponding group fed a control diet (*n* = 6 mice/group).

Table S1 Fatty acids content in control and fish oil-enriched diet.
