# Supplementary figures and images for: Anxiolytic- and Antidepressant-Like Effects of Fish Oil-Enriched Diet in Brain-Derived Neurotrophic Factor Deficient Mice
Source: Front Neurosci. 2018 Dec 21;12:974. doi: 10.3389/fnins.2018.00974 (PMC6308198; doi:10.3389/fnins.2018.00974)

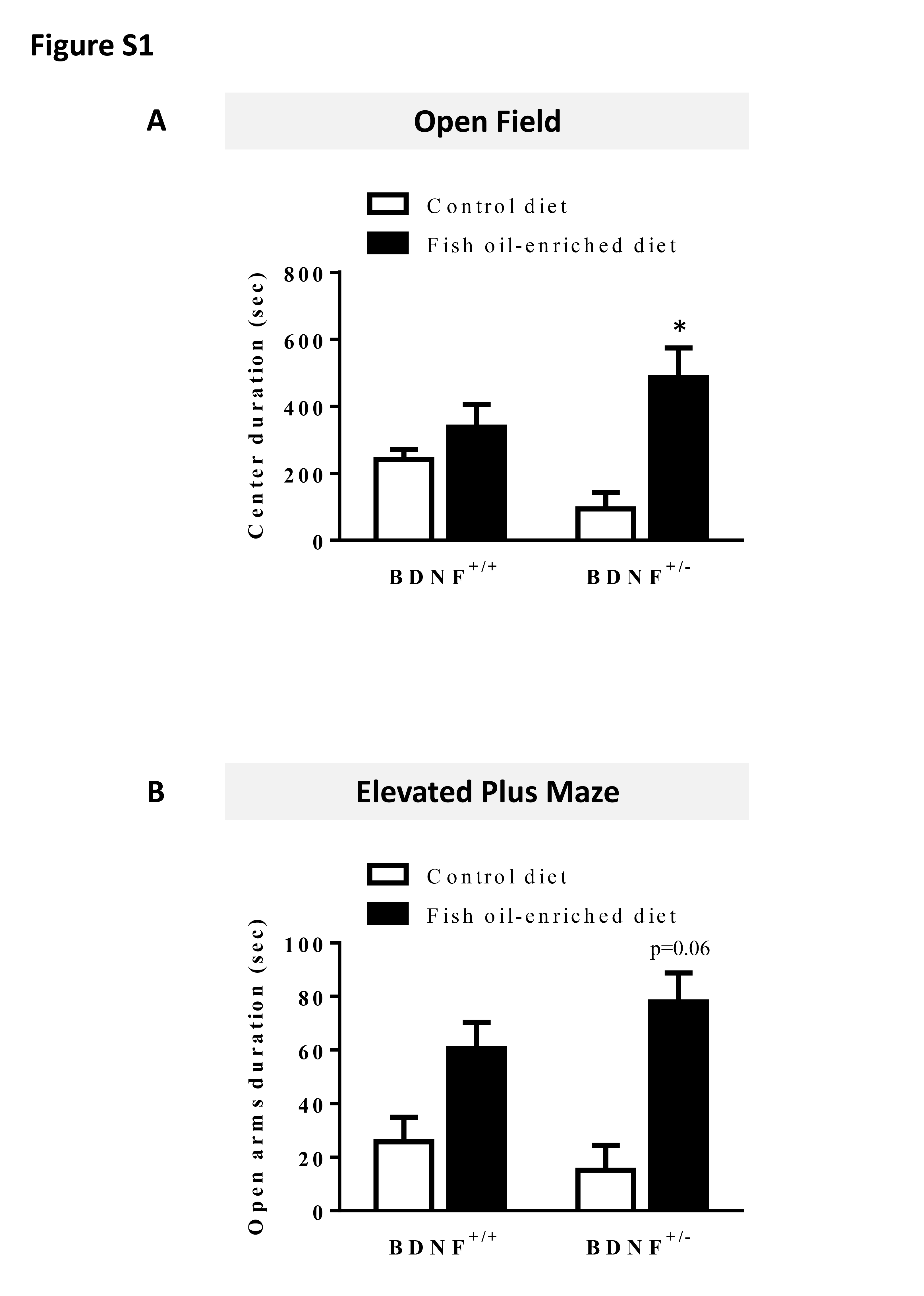

Supplement: Figure S1 — Fish oil-enriched diet induces anxiolytic-like activities in BDNF+/- mice. (A) Anxiety evaluated in the open field. Data are means ± SEM of the time spent in the center of the arena (ANOVA: [F(3,20) = 7.1; p = 0.002]). (B) Anxiety evaluated in the elevated plus maze. Data are means ± SEM of the time spent in the open arms (ANOVA: [F(3,20) = 2.8; p = 0.11]). ∗p < 0.05: significantly different from the corresponding group fed a control diet (n = 6 mice/group). [file Image_1.tif]
