# Supplementary material for: Anxiolytic- and Antidepressant-Like Effects of Fish Oil-Enriched Diet in Brain-Derived Neurotrophic Factor Deficient Mice
Source: Front Neurosci. 2018 Dec 21;12:974. doi: 10.3389/fnins.2018.00974 (PMC6308198; doi:10.3389/fnins.2018.00974)
Supplement: Table S1 — Fatty acids content in control and fish oil-enriched diet. [file Table_1.DOCX]

**Supplemental Table 1.** Fatty acids content in control and fish oil-enriched diet.

| **Fatty acids** | **Control diet** | **Fish oil-enriched diet** |
| --- | --- | --- |
| C14:0 | 0.51 | 7.54 |
| C16:0 | 23.18 | 20.34 |
| C18:0 | 7.27 | 3.57 |
| **∑ SFA** | **31.59** | **32.28** |
| C16:1 cis-9 | 0.04 | 0.28 |
| C18:1 cis-9 | 39.82 | 10.24 |
| **∑ MUFA** | **41.92** | **26.35** |
| C18:2 n-6 (LA) | 12.88 | 8.07 |
| C20:4 n-6 (AA) | 0.04 | 1.04 |
| C18:3 n-3 (ALA) | 12.28 | 2.63 |
| C20:5 n-3 (DPA) | 0.50 | 14.17 |
| C22:5 n-3 (EPA) | 0.01 | 2.06 |
| C22:6 n-3 (DHA) | 0.12 | 9.16 |
| **∑ PUFAs** | **26.49** | **41.37** |
| ∑ PUFAs omega-6 | 12.98 | 9.38 |
| ∑ PUFAs omega-3 | 13.51 | 31.99 |
| ômega-6/omega-3 | 0.961 | 0.293 |
